# Neoadjuvant or adjuvant therapy for resectable esophageal cancer: a systematic review and meta-analysis

**DOI:** 10.1186/1741-7015-2-35

**Published:** 2004-09-24

**Authors:** Richard A Malthaner, Rebecca KS Wong, R Bryan Rumble, Lisa Zuraw, Members of the Gastrointestinal Cancer Disease Site Group of Cancer Care Ontario's Program in Evidence-based Care.

**Affiliations:** 1 University of Western Ontario, London Health Sciences Centre Division of Thoracic Surgery and Surgical Oncology, London, Ontario, Canada; 2 Princess Margaret Hospital, University of Toronto, Toronto, Ontario, Canada; 3 Department of Clinical Epidemiology & Biostatistics, McMaster University, Hamilton, Ontario, Canada

## Abstract

**Background:**

Carcinoma of the esophagus is an aggressive malignancy with an increasing incidence. Its virulence, in terms of symptoms and mortality, justifies a continued search for optimal therapy. The large and growing number of patients affected, the high mortality rates, the worldwide geographic variation in practice, and the large body of good quality research warrants a systematic review with meta-analysis.

**Methods:**

A systematic review and meta-analysis investigating the impact of neoadjuvant or adjuvant therapy on resectable thoracic esophageal cancer to inform evidence-based practice was produced.

MEDLINE, CANCERLIT, Cochrane Library, EMBASE, and abstracts from the American Society of Clinical Oncology and the American Society for Therapeutic Radiology and Oncology were searched for trial reports.

Included were randomized trials or meta-analyses of neoadjuvant or adjuvant treatments compared with surgery alone or other treatments in patients with resectable thoracic esophageal cancer. Outcomes of interest were survival, adverse effects, and quality of life. Either one- or three-year mortality data were pooled and reported as relative risk ratios.

**Results:**

Thirty-four randomized controlled trials and six meta-analyses were obtained and grouped into 13 basic treatment approaches.

Single randomized controlled trials detected no differences in mortality between treatments for the following comparisons:

- Preoperative radiotherapy versus postoperative radiotherapy.

- Preoperative and postoperative radiotherapy versus postoperative radiotherapy. Preoperative and postoperative radiotherapy was associated with a significantly higher mortality rate.

- Postoperative chemotherapy versus postoperative radiotherapy.

- Postoperative radiotherapy versus postoperative radiotherapy plus protein-bound polysaccharide versus chemoradiation versus chemoradiation plus protein-bound polysaccharide.

Pooling one-year mortality detected no statistically significant differences in mortality between treatments for the following comparisons:

- Preoperative radiotherapy compared with surgery alone (five randomized trials).

- Postoperative radiotherapy compared with surgery alone (five randomized trials).

- Preoperative chemotherapy versus surgery alone (six randomized trials).

- Preoperative and postoperative chemotherapy versus surgery alone (two randomized trials).

- Preoperative chemoradiation therapy versus surgery alone (six randomized trials).

Single randomized controlled trials detected differences in mortality between treatments for the following comparison:

- Preoperative hyperthermia and chemoradiotherapy versus preoperative chemoradiotherapy in favour of hyperthermia.

Pooling three-year mortality detected no statistically significant difference in mortality between treatments for the following comparison:

- Postoperative chemotherapy compared with surgery alone (two randomized trials).

Pooling three-year mortality detected statistically significant differences between treatments for the following comparisons:

- Preoperative chemoradiation therapy versus surgery alone (six randomized trials) in favour of preoperative chemoradiation with surgery.

- Preoperative chemotherapy compared with preoperative radiotherapy (one randomized trial) in favour of preoperative radiotherapy.

**Conclusion:**

For adult patients with resectable thoracic esophageal cancer for whom surgery is considered appropriate, surgery alone (i.e., without neoadjuvant or adjuvant therapy) is recommended as the standard practice.

## Background

Carcinoma of the esophagus is an aggressive malignancy that continues to kill more than 90% of people with the disease within five years [1]. The incidence of adenocarcinoma of the esophagus is rising faster than any other malignancy [2]. In 2001, there were at least 1,450 deaths due to esophageal cancer in Canada and many more people suffered because of the disease [3]. Its virulence, in terms of symptoms and mortality, justifies a continued search for optimal therapy.

Surgical esophagectomy remains the preferred treatment for clinically localized thoracic esophageal carcinoma [1,4-6]. Two randomized trials comparing surgery alone to radiation alone found surgery to be the better treatment for resectable cancer [5,6]. Fok et al randomly assigned 39 patients to surgery and 35 patients to 45 to 53 Gy radiation over four to five weeks [5]. The median survival time and five-year survival rate for surgery were 21.6 months and 16%, respectively, compared with 8.2 months and 7% for radiation (p < 0.05). Badwe et al compared 47 surgical patients to 52 patients undergoing 50 Gy radiation in 28 fractions plus 15 Gy boost in 8 fractions or 15 Gy brachytherapy [6]. Overall survival was better with surgery (odds ratio [OR], 2.74; 95% confidence interval [CI], 1.51 to 4.98; log-rank p = 0.002). The swallowing status was better in the surgery arm at six months after treatment (p = 0.03). Survival data from these two trials were pooled. The pooled results favoured surgery alone. There was no statistical heterogeneity (X^2 ^= 0.02, p = 0.9) and a 52% relative increase in the risk of death at three years with radiotherapy compared with surgery alone (relative risk ratio [RR], 1.52; 95% CI, 1.23 to 1.86; p = 0.0007).

The failure of surgery alone is attributed to the systemic nature of the disease at the time of presentation [7,8]. Early and effective systemic chemotherapy and local radiotherapy, directed at micro-metastases and added to surgical resection, could lead to increased survival. Many clinical trials have evaluated the role of adjuvant therapy, both preoperatively and postoperatively, with conflicting results. Patients with cervical esophageal cancer are generally treated with chemoradiation, either preoperatively or postoperatively, in an attempt to avoid a laryngoesophagectomy and preserve the larynx. Although the majority of studies have been performed in squamous cell carcinomas, adenocarcinomas were included in some studies, but a distinction between the two histological subtypes was not made in this guideline report because previous studies have not consistently found that they respond differently to chemotherapy or radiation [9-17].

The large and growing number of patients affected, the high mortality rates, the geographic variation in practice, and the large body of good quality research evidence warrants a systematic review with meta-analysis.

## Methods

This systematic review was developed by the Practice Guidelines Initiative (PGI) of Cancer Care Ontario's Program in Evidence-based Care (PEBC). Evidence was selected and reviewed by two members of the PGI's Gastrointestinal Cancer Disease Site Group (DSG) and two methodologists. Members of the Gastrointestinal Cancer DSG disclosed potential conflict of interest information.

This systematic review is a convenient and up-to-date source of the best available evidence on neoadjuvant or adjuvant therapy for resectable esophageal cancer. The body of evidence in this systematic review is primarily comprised of mature randomized controlled trial data; it forms the basis of a clinical practice guideline developed by the Gastrointestinal Cancer DSG published elsewhere (18). This systematic review and companion practice guideline are intended to promote evidence-based practice in Ontario, Canada. The PGI is editorially independent of Cancer Care Ontario and the Ontario Ministry of Health and Long-Term Care.

### Literature search strategy

The MEDLINE (1966 through October (week 2) 2003), CANCERLIT (1983 to October 2001), Cochrane Library (2003, Issue 3), and EMBASE (to week 40, 2003) databases were searched with no language restrictions. "Esophageal neoplasms" (Medical subject heading (MeSH)) was combined with "chemotherapy, adjuvant" (MeSH), "radiotherapy, adjuvant" (MeSH), "immunotherapy, adjuvant" (MeSH), and each of the following phrases used as text words: "preoperative", "neoadjuvant", "chemotherapy", "radiotherapy", "radiation therapy", "irradiation", "immunotherapy", "chemoradiotherapy", "chemoradiation", and "hyperthermia". These terms were then combined with the search terms for the following study designs or publication types: practice guidelines, meta-analyses, and randomized controlled trials. Additionally, the conference proceedings of the 1997 to 2003 annual meetings of the American Society of Clinical Oncology (ASCO) and the 1999 to 2002 annual meetings of the American Society for Therapeutic Radiology and Oncology (ASTRO) were searched for reports of new or ongoing trials. Relevant articles and abstracts were reviewed, and the reference lists from these sources were searched for additional trials. This formal search was supplemented with published abstracts from thoracic surgery and oncology conferences, conversations with colleagues and experts in the field, and a review of textbooks related to esophageal oncology.

### Study selection criteria

Articles were included in this systematic review if they were fully published reports, abstracts, or meta-analyses of randomized controlled trials (RCT) of neoadjuvant or adjuvant treatments compared with surgery alone or surgery plus another treatment in patients with resectable and operable thoracic esophageal cancer. Data on survival had to be reported. Other outcomes of interest were adverse effects and quality of life. Reports of carcinomas located in the cervical esophagus were excluded.

### Synthesizing the evidence

Because diverse treatment strategies were evaluated, the eligible studies were grouped into 13 basic treatment approaches (Table 1), and each group was examined separately. Pooling was conducted using one-year mortality data for all meta-analyses except for the comparison of post-operative chemotherapy versus surgery alone, which was pooled at three years. Any time point selected for meta-analyses must be clinically credible and relevant but not so far along the survival curve that wide confidence intervals result from fewer patients contributing to the estimate. Since time points prior to the median will generally ensure that there are sufficient data to be credible, median survival times, weighted by the size of the treatment arms, were calculated to determine the time point for each meta-analysis as recommended in the literature [19]. Studies that did not provide values for survival at the time of pooling were not included in each meta-analysis, although they were included in calculating the weighted median survival time, if values for median survival were provided. All pooling was performed with Review Manager 4.2.1, available through the Cochrane Collaboration [Review Manager (RevMan) [Computer program]. Version 4.2 for Windows. Oxford, England: The Cochrane Collaboration, 2003]. Pooled results were expressed as mortality RR with 95% CI using the random effects model. An RR less than 1.0 favours neoadjuvant or adjuvant treatment, and an RR greater than 1.0 favours surgery alone. All analyses were made based on the intent-to-treat principle, except where only evaluable patient data were available.

### Potential sources of heterogeneity and sensitivity analysis

Heterogeneity of study results was assessed using a visual plot of the outcomes and by calculating the X^2 ^(Chi-square) statistic using a planned cut-off for significance of p < 0.05. Potential sources of heterogeneity were postulated a priori and included study quality assessed with the Jadad scale [20] (>2 versus ≤2), full article publication versus abstract publication, squamous cell versus adenocarcinoma, type of chemotherapy (cisplatin-containing versus others), type of surgery (transthoracic versus transhiatal), and radiotherapy dose (BED>48 versus BED<48). To facilitate comparison across trials, radiotherapy dose was converted to biological equivalent dose (same as biological effective dose) using the equation BED = nd (1+d/α/β), where n = number of fractions, d = dose per fraction, and it is assumed that α/β = 10 for tumour effect. Due to limitations inherent with this model, no allowances can be made for any time gaps in split-course treatments. These factors were used to explore any significant heterogeneity of results across the trials. The robustness of our conclusions was examined through subsequent sensitivity analyses using these factors. The sensitivity analysis results are not detailed, as they would not change the conclusions.

## Results

### Literature search results

Thirty-four randomized controlled trials were obtained. Of these, 30 were fully published reports [5,21-25,27-34,36,37,39,40,42-48,52,56-60], and four were available in abstract form only [35,41,51,53]. The four-arm trials by Fok et al [5] and Nygaard et al [24] contributed to multiple comparisons. Additionally, six meta-analyses were obtained, five fully published [26,38,49,50,55] and one abstract [54]. Literature search results appear in Table 1.

## Outcomes

### Preoperative radiotherapy and surgery versus surgery alone

**Figure 1:**
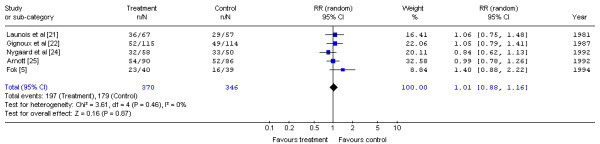
**Meta-analysis examining preoperative radiotherapy and surgery compared to surgery alone: mortality at one year. **Overall risk ratio = 1.01 (95% CI, 0.88 to 1.16; p = 0.87)

Six randomized trials of preoperative radiotherapy and surgery versus surgery alone are presented in Table 2[5,21-25]. The radiotherapy regimens varied, using low to moderate doses ranging from 20 Gy in 10 fractions to 53 Gy in 20 fractions. Treatment was delivered between one to four weeks prior to surgery. Quality-of-life assessments were not conducted in any of the six trials. The Gastrointestinal Cancer DSG pooled the five trials that reported one-year mortality data [5,21,22,24,25] (Figure 1). No statistically significant difference in the risk of mortality with preoperative radiotherapy at one year compared with surgery alone was detected (RR, 1.01; 95% CI, 0.88 to 1.16; p = 0.87). No statistical heterogeneity was detected (X^2 ^= 3.61, p = 0.46).

A published meta-analysis [26] using updated individual patient data on 1147 patients from five trials [21-25] detected a hazard ratio for death of 0.89 (95% CI, 0.78 to 1.01; p = 0.062) for preoperative radiotherapy compared with surgery alone. This meta-analysis included additional patients from the study by Wang et al. [23] with no description of why these patients were excluded from the published report of the trial (a total of 418 patients from this study were included in the meta-analysis versus 206 included in the trial report). The trial by Fok et al. [5] was not included in the published meta-analysis.

### Postoperative radiotherapy and surgery versus surgery alone

Five randomized trials of surgery and postoperative radiotherapy compared with surgery alone are presented in Table 3[5,27-29,47]. Although all studies specified the absence of distant metastases as an inclusion criterion, Zieren et al. [29] and Teniere et al. [28] included patients with celiac node involvement (M1 disease). Fok et al. [27] included patients with positive margins and "a high chance of residual tumour". In the trials by Fok et al., Zieren et al. and Xiao et al., radiotherapy was delivered within six weeks postoperatively, while the trial by Teniere et al. specified within three months. The radiotherapy doses were higher than in the preoperative series. Of note, Fok et al. employed hypofractionation schedules using three fractions per week and 3.5 Gy per fraction to total doses of 49 Gy for patients with negative margins and 52.5 Gy for those with positive margins.

Only Zieren et al. assessed quality of life. The results indicated more rapid recovery of quality of life with surgery alone compared with postoperative radiotherapy. Three trials [28,29,47] demonstrated no significant difference in survival while another [27] found significantly shorter survival with postoperative radiotherapy and surgery compared with surgery alone.

**Figure 2:**
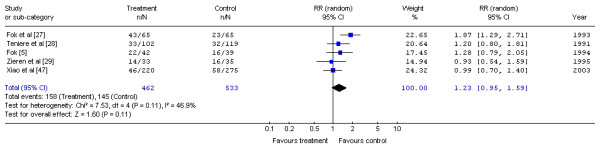
**Meta-analysis examining postoperative radiotherapy and surgery compared to surgery alone: mortality at one year. **Overall risk ratio = 1.23 (95% CI, 0.95 to 1.59; p = 0.11)

The Gastrointestinal Cancer DSG pooled the five trials that reported one-year mortality data [5,27-29,47] (Figure 2). No significant difference in the risk of mortality with postoperative radiotherapy and surgery at one year compared with surgery alone was detected (RR, 1.23; 95% CI, 0.95 to 1.59; p = 0.11). No significant statistical heterogeneity was detected (X^2 ^= 7.53, p = 0.11). The rate of local recurrence with radiotherapy was lower in three of the trials [27,28,47], but two trials [27,28] noted this benefit was achieved at the expense of increased morbidity.

### Preoperative radiotherapy versus postoperative radiotherapy

One randomized trial evaluated preoperative radiotherapy versus postoperative radiotherapy with curative esophagectomy as part of a four-arm study [5]. Patients in this trial, performed between 1968 and 1981, received from 24 to 53 Gy preoperatively (n = 40) or 45 to 53 Gy postoperatively (n = 42). The median survival was 11 months for both groups. No difference in the survival rate was detected, but there was increased morbidity with preoperative radiotherapy. Quality of life was not assessed in this trial.

### Preoperative radiotherapy and postoperative radiotherapy versus postoperative radiotherapy alone

Iizuka et al. [30] reported a randomized trial of preoperative and postoperative radiotherapy versus postoperative radiotherapy alone in 364 Japanese patients. In an analysis of 207 eligible patients (157 patients were excluded because of the extent of disease or operative complications), preoperative and postoperative radiotherapy was associated with a significantly higher mortality rate compared with postoperative radiotherapy alone (median survival was 394 days versus 648 days; p = 0.0069). The major postoperative complications were pneumonia (13.5% versus 9.7%) and leakage (11.5% versus 9.7%).

### Preoperative chemotherapy and surgery versus surgery alone

Seven randomized trials of preoperative chemotherapy and surgery versus surgery alone are presented in Table 4[24,32-35,37,48]. Of these seven RCTs, six were available as fully published reports, and one was available as an abstract only [35]. Quality of life was not assessed in any of the trials. Additionally, three meta-analyses were obtained [38,49,50].

**Figure 3:**
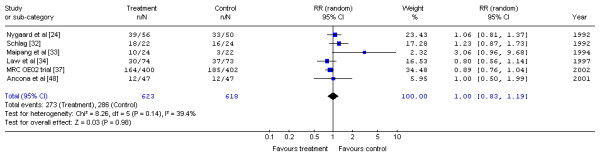
**Meta-analysis examining preoperative chemotherapy and surgery compared to surgery alone: mortality at one year. **Overall risk ratio = 1.00 (95% CI, 0.83 to 1.19; p = 0.98)

The Gastrointestinal Cancer DSG pooled the available data on preoperative chemotherapy with surgery versus surgery alone [24,32-34,37,48] (Figure 3). No significant difference in the risk of mortality at one year was detected (RR, 1.00; 95% CI, 0.83 to 1.19; p = 0.98). No statistical heterogeneity was detected (X^2 ^= 8.26, p = 0.14).

The first meta-analysis, by Bhansali et al. [38], pooled data from 12 randomized trials of chemotherapy in a variety of combinations with radiotherapy with and without surgery, and no benefit for cisplatin-based chemotherapy was detected (OR, 0.96; 95% CI, 0.75 to 1.22; p > 0.10). This published meta-analysis included only four of the eight trials of preoperative chemotherapy versus surgery alone [24,31-33]. Trials that did not meet the inclusion criteria for this systematic review, such as trials involving patients with inoperable esophageal cancer and trials of combined chemoradiotherapy versus radiotherapy alone in the non-surgical management of esophageal cancer, were included in the Bhansali et al. meta-analysis.

The second meta-analysis, by Urschel et al. [49], pooled data from 11 RCTs (a total of 1,976 patients). These 11 RCTs were graded for quality using the Jadad scale [20]. Pooling detected no statistically significant difference between combination preoperative chemotherapy with surgery over surgery alone for survival at either one year (OR 1.00; 95% CI 0.76–1.30; p = 0.98), two years (OR 0.88; 95% CI 0.62–1.24; p = 0.45), or three years (OR 0.77; 95% CI 0.37–1.59; p = 0.48).

The third meta-analysis [50] was a Cochrane Review which pooled 11 RCTs (a total of 2,051 patients). Survival RRs were calculated at one, two, three, four, and five years, but a statistically significant difference in survival favouring preoperative chemotherapy was detected only at five years (RR = 1.44, 95% CI; 1.05–1.97; p = 0.02).

### Preoperative and postoperative chemotherapy and surgery versus surgery alone

**Figure 4:**
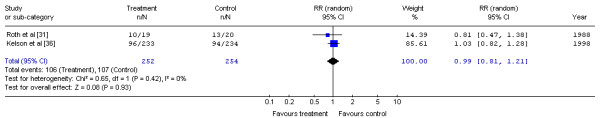
**Meta-analysis examining preoperative and postoperative chemotherapy and surgery to surgery alone: mortality at one year. **Overall risk ratio = 0.99 (95% CI, 0.81 to 1.21; p = 0.93)

Two randomized trials of preoperative and postoperative chemotherapy and surgery versus surgery alone [31,36] (Table 5) were examined. Neither Roth et al. [31] (using a now out-dated combination of cisplatin, vindesine, and bleomycin) nor the largest North American trial as reported by Kelsen et al. [36] (using cisplatin and 5-FU) detected a statistically significant difference in overall survival. The Gastrointestinal Cancer DSG pooled these two trials (Figure 4). No significant difference in the risk of mortality with preoperative and postoperative chemotherapy and surgery compared with surgery alone was detected (RR, 0.99; 95% CI, 0.81 to 1.21; p = 0.93). No statistical heterogeneity was detected (X^2 ^= 0.65, p = 0.42).

### Postoperative chemotherapy and surgery versus surgery alone

Three randomized trials of postoperative chemotherapy and surgery compared with surgery alone are presented in Table 6[39-41]. All three trials used cisplatin-based regimens. Pouliquen et al. [39] found no improvement in the survival rate with postoperative chemotherapy. The patients were stratified into two groups: complete resections with or without nodal involvement and, palliative resections for positive margins or metastatic disease. Only the completely resected group was included in our analysis. Ando et al. [40] resected early (T1b) carcinomas and did not find any improvement in survival. In another study, reported in abstract form, Ando et al. [41] also found no survival benefit for postoperative chemotherapy in localized squamous cell carcinoma of the thoracic esophagus. Pouliquen et al. assessed quality of life and found that the duration of improved dysphagia was similar for both groups.

**Figure 5:**
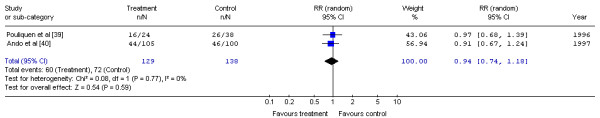
**Meta-analysis examining postoperative chemotherapy and surgery compared to surgery alone: mortality at three years. **Overall risk ratio = 0.94 (95% CI, 0.74 to 1.18; p = 0.59)

The Gastrointestinal Cancer DSG pooled the three-year mortality data for two trials [39,40] (Figure 5). The trial by Ando et al. [41] could not be included in the pooled analysis because the abstract did not report three-year survival. No significant difference in the risk of mortality at three years for postoperative chemotherapy compared with surgery alone was detected (RR, 0.94; 95% CI, 0.74 to 1.18; p = 0.59). There was no significant statistical heterogeneity (X^2 ^= 0.08, p = 0.77).

### Preoperative chemotherapy and radiotherapy and surgery versus surgery alone

Eight randomized trials of combined modality neoadjuvant chemotherapy and radiotherapy are presented in Table 7[24,42-46,51,53]. A ninth trial obtained [52] provided updated five-year data for another report [44]. None of the trials reported data on quality of life. In contrast to the other trials, the study by Walsh et al. [44,52] reported a significant overall increase in three-year survival with combined preoperative chemoradiation but was closed prematurely following an interim analysis. This study was criticized for the lack of preoperative staging using CT scans, premature closure, and an unusually poor survival rate in the surgery-alone arm.

**Figure 6:**
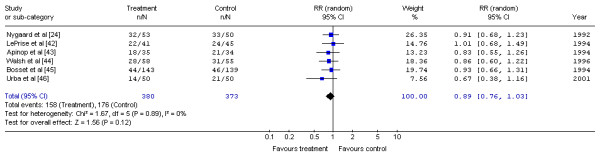
**Meta-analysis examining preoperative chemoradiation and surgery compared to surgery alone: mortality at one year. **Overall risk ratio = 0.89 (95% CI, 0.76 to 1.03; p = 0.12)

The Gastrointestinal Cancer DSG pooled the one-year mortality data for the six trials with data available at one year [24,42-46] (Figure 6). No significant difference in the risk of mortality at one year for preoperative chemoradiation and surgery compared to surgery alone was detected (RR, 0.89; 95% CI, 0.76 to 1.03; p = 0.12). No significant statistical heterogeneity was detected (X^2 ^= 1.67, p = 0.89).

The first meta-analysis, an abstract report by Fiorica et al. [54], pooled six RCTs comparing preoperative chemoradiation and surgery versus surgery alone. A systematic review, restricted to trials that included only patients with resectable esophageal carcinoma with no metastatic disease, obtained six RCTs. A significant difference in three-year mortality favouring neoadjuvant therapy with surgery versus surgery alone was detected (OR 0.53; 95% CI 0.31–0.92; p = 0.025). A conclusion was made that neoadjuvant chemoradiation and surgery significantly improved three-year survival compared to surgery alone in patients with resectable esophageal cancer but acknowledged that the magnitude of the benefit was relatively small. The authors recommend that research to determine the criteria that would identify patients likely to benefit from neoadjuvant chemoradiation be undertaken.

The second meta-analysis, by Urschel et al. [55], pooled nine RCTs, eight of which were included in this practice guideline [24,42-46,51,52]. The RCTs were graded for quality using the Jadad scale. This meta-analysis did not find a statistically significant difference in mortality at one year (OR 0.79; 95% CI 0.59–1.06; p = 0.12) or at two years (OR 0.77; 95% CI 0.59–1.05; p = 0.10). However, as in the meta-analysis by Fiorica et al. [54], a statistically significant difference was found at three years in favour of preoperative chemoradiation (OR 0.66; 95% CI 0.47–0.92; p = 0.016). The authors noted that the three-year survival benefit was most pronounced when chemoradiation was given concurrently (OR 0.45; 95% CI 0.26–0.79; p = 0.005) as opposed to sequentially (OR 0.82; 95% CI 0.54–1.25; p = 0.36).

To compare the results between the two published meta-analyses [54,55] with the trials included in this systematic review, the Gastrointestinal Cancer DSG pooled the data comparing neoadjuvant chemoradiation with surgery versus surgery alone at three years and obtained similar results. A significant difference in the risk of mortality at three years favouring neoadjuvant chemoradiation with surgery versus surgery alone was detected (RR = 0.87; 95% CI 0.80–0.96; p = 0.004). No statistically significant heterogeneity was detected (X^2 ^= 6.59, p = 0.25).

### Postoperative chemotherapy and radiotherapy versus surgery alone

No randomized trials have evaluated postoperative chemotherapy combined with radiation versus surgery alone.

### Postoperative chemotherapy versus postoperative radiotherapy

One randomized trial evaluated postoperative chemotherapy versus postoperative radiotherapy following curative esophagectomy [56]. Patients in this Japanese trial received cisplatin and vindesine (n = 126) or radiotherapy at a dose of 50 Gy (n = 127). The median survival was 38 months for both groups. No difference in survival was detected (52% for chemotherapy versus 51% for radiotherapy at three years; log-rank p = 0.806). There were significantly more cases of decreased white blood cell counts (12 versus 3 for grade 3–4; p = 0.026), elevated blood urea nitrogen (26 versus 11 for grade 1–2; p = 0.018) and elevated creatinine concentrations (27 versus 9 for grade 1–3; p = 0.006) among patients randomized to chemotherapy compared with radiotherapy. Quality of life was not assessed in this trial.

### Preoperative chemotherapy versus preoperative radiotherapy

Two randomized trials evaluating preoperative chemotherapy compared with preoperative radiotherapy were reviewed [24,57]. Kelsen et al. [57] randomly assigned 96 patients to preoperative radiotherapy or chemotherapy. Postoperative crossover therapy (i.e., postoperative radiotherapy for those who received preoperative chemotherapy and vice versa) was given to patients who were found to have unresectable or locally advanced disease. Only 11 of 48 chemotherapy patients and 9 of 48 radiotherapy patients did not receive additional postoperative treatment. Overall median survival was similar in both groups (10.4 months for chemotherapy versus 12.4 months for radiotherapy; p = 0.61), but the crossover design precluded proper analysis. In the four-arm trial by Nygaard et al. [24], preoperative chemotherapy was compared with preoperative radiotherapy, and the results demonstrated a significant difference in survival favouring preoperative radiotherapy (21% versus 3% at three years; p = 0.01). However, when compared to surgery alone, there was no benefit to either preoperative radiation or chemotherapy. Neither trial report included data on quality of life.

### Preoperative chemoradiation versus preoperative radiotherapy

One randomized trial evaluated the role of preoperative bleomycin in addition to radiotherapy [58]. Seventy patients received preoperative chemoradiation with bleomycin and 63 patients received preoperative radiotherapy alone. The results demonstrated no significant difference in survival between the two groups (median survival was 25 weeks versus 26 weeks; survival rate was 25% versus 19% at two years; p = 0.56). There was also no benefit for bleomycin in the palliation of dysphagia. Quality of life was not assessed in this trial.

### Postoperative immunotherapy in combination with radiotherapy or chemoradiation

One Japanese trial evaluated protein-bound polysaccharide (PSK) as an adjunct to postoperative radiotherapy or chemoradiation in resected esophageal cancer [59]. This trial involved 174 patients who were randomly assigned to four treatment groups. The three-year survival rates for radiotherapy, radiotherapy + PSK, chemoradiotherapy, and chemoradiotherapy + PSK were 43.3%, 45.5%, 33.5%, and 44.3%, respectively. There was no significant difference in survival when radiotherapy and radiotherapy + PSK were compared, or when chemoradiotherapy and chemoradiotherapy + PSK were compared (log-rank p = 0.19 for chemoradiotherapy versus chemoradiotherapy + PSK). Some patients randomized to PSK experienced adverse effects, including mild nausea, erythema, liver dysfunction and leukopenia, but there were no reports of toxicity that were definitely attributed to PSK. There was no assessment of quality of life.

### Preoperative hyperthermia in combination with chemoradiation

One Japanese randomized trial, reported by Kitamura et al. [60], evaluated preoperative hyperthermia and chemoradiotherapy and surgery (n = 32) versus preoperative chemoradiotherapy and surgery (n = 34). Median survival was 36 months and 20 months, respectively. The results showed a significant improvement in the survival rate (50.4% versus 24.2% at three years; p-value not reported) and local tumour control with hyperthermia compared with control. It was reported that both adjuvant treatments were well tolerated and resulted in no postoperative complications. Quality of life was not assessed.

### Adverse effects

Adverse effects were inconsistently reported (Tables 2,3,4,5,6,7). Most patients experienced treatment-related adverse effects associated with radiotherapy or chemotherapy.

## Discussion

Most trials excluded patients with cancers located in the cervical esophagus, and therefore the interpretation of this review is limited to tumours in the more distal two thirds.

The options for neoadjuvant or adjuvant therapy for resectable thoracic esophageal cancer are many. On reviewing the results of randomized trials and meta-analyses, the Gastrointestinal Cancer DSG did not recommend neoadjuvant or adjuvant therapy based on the following:

Preoperative radiotherapy does not improve survival compared with surgery alone. Postoperative radiotherapy may, in fact, be harmful [27].

Preoperative chemoradiation does not appear to improve survival compared to surgery alone. Although the pooled analysis shows that all six studies are in the direction favouring preoperative chemoradiation at one year, the pooled estimate did not achieve statistical significance. When examining the individual trial results, only the trial by Walsh et al. [44] detected a statistically significant survival benefit, but this trial has been criticized for its methodology. The most recent trials, conducted by Bosset et al. [45] and Urba et al. [46], have five-year data available, and neither detected a statistically significant difference in survival between preoperative chemoradiation and surgery alone.

Preoperative cisplatin-based chemotherapy does not appear to improve survival. Four of the seven trials [24,32-34] detected a significant survival advantage favouring preoperative cisplatin-based chemotherapy. Kok et al. [35] reported a survival advantage for chemotherapy but only reported median survival results in abstract form. The two largest trials produced conflicting results [36,37]. Kelsen et al. [36] detected no survival advantage, while the MRC OE02 trial [37] detected a significant survival advantage for preoperative chemotherapy at two years. Although all chemotherapy protocols were cisplatin-based, the varying dosages, the number of cycles completed, and the other agents used contributed to clinical heterogeneity.

The available evidence from three randomized trials does not support the use of postoperative chemotherapy over surgery alone [39-41].

Two novel approaches, immunotherapy and hyperthermia, were studied by two groups in Japan [59,60]. Ogoshi et al. [59] detected no significant survival benefit for patients treated with PSK versus without PSK. Although Kitamura et al. [60] found a significant improvement in survival and local control favouring preoperative chemoradiotherapy with hyperthermia versus without hyperthermia, the Gastrointestinal Cancer DSG felt the results should be interpreted with caution until further confirmatory trials are conducted.

Examination of the results of randomized trials, including the pooled analyses, fails to support the use of preoperative or postoperative adjuvant treatment of any type at this time for patients with resectable carcinoma of the thoracic esophagus. Overall, the evidence does not support the use of neoadjuvant or adjuvant therapy for patients with resectable cancer of the lower two-thirds of the esophagus. Surgical resection alone should remain the standard for localized thoracic esophageal cancer. Patient staging information can be found in Appendix 1 (Additional file 1).

Future trials should continue to assess multi-modality treatments for this patient population. For clinicians seeking guidance on treatments for patients with non-resectable esophageal cancer, the role of radiotherapy alone and chemoradiation alone without surgery is addressed in another clinical practice guideline by the Gastrointestinal Cancer DSG, *Combined modality radiotherapy and chemotherapy in the non-surgical management of localized carcinoma of the esophagus *[61].

## List of abbreviations used

In order of appearance:

Gy, Gray; OR, odds ratio; DSG, Disease Site Group; RR, (relative) risk ratio; PGI, Practice Guidelines Initiative; PEBC, Program in Evidence-based Care; MeSH, Medical subject heading; ASCO, American Society of Clinical Oncologists; ASTRO, American Society for Therapeutic Radiology and Oncology; RCT, randomized controlled trial; CI, confidence interval; X^2^, Chi-square; BED, biological equivalent (or effective) dose; PSK, protein-bound polysaccharide; MRC, Medical Research Council; PGCC, Practice Guidelines Coordinating Committee.

## Competing interests

The author(s) declare that they have no competing interests.

## Authors' contributions

RM, RW and LZ performed the initial literature search and created the initial drafts of the systematic review with input from other members of the Gastrointestinal Cancer DSG. RM, RW and BR created the final draft of this systematic review. All statistical analysis was performed by BR in consultation with RM and RW. Creation of the submitted manuscript was performed by BR and RM.

## Pre-publication history

The pre-publication history for this paper can be accessed here:



## Supplementary Material

Additional file 1 Click here for file

## Figures and Tables

**Table 1:** Studies included in this systematic review.

**Treatment Approach**	**Number of Trials**	**Reference Numbers**	**Summary of Results**
**Randomized Controlled Trials**			
Preoperative RT v. Surgery Alone	6	5*,21–25†	Table 2
Postoperative RT v. Surgery Alone	4	5*,27–29,47	Table 3
Preoperative RT v. Postoperative RT	1	5*	-
Preoperative RT + Postoperative RT v. Postoperative RT	1	30	-
Preoperative CT v. Surgery Alone	6	24†,32–35^‡^,37,48	Table 4
Preoperative + Postoperative CT v. Surgery Alone	2	31,36	Table 5
Postoperative CT v. Surgery Alone	3	39–41^‡^	Table 6
Preoperative CRT v. Surgery Alone	6	24†,42–46,51–53^‡^	Table 7
Postoperative CT v. Postoperative RT	1	56	-
Preoperative CT v. Preoperative RT	2	24†,57	-
Preoperative CRT v. Preoperative RT	1	58	-
Postoperative Immunotherapy with RT or CRT v. RT or CRT	1	59	-
Preoperative Hyperthermia with CRT v. preoperative CRT	1	60	-
**Meta-analyses**			
Preoperative RT v. Surgery Alone	1	26	-
Preoperative CT v. Surgery Alone	2	38,49,50	-
Preoperative CRT v. Surgery Alone	2	54^‡^,55	-

Note: CT indicates chemotherapy; CRT, chemoradiation; RT, radiotherapy; v., versus.

* The four-arm trial by Fok et al [5] contributed to three comparisons.

† The four-arm trial by Nygaard et al [24] contributed to four comparisons.

‡ Reports published in abstract form only [35,41,51,53,54].

**Table 2:** Randomized trials of preoperative radiotherapy (RT) and surgery versus surgery alone.

**Study, year [Reference]**	**Participants**	**Number of patients**	**Interventions**	**Median Survival (Months)**	**Survival Rate (%)**					**Adverse Effects (Number of Patients)**
					**1 yr**	**2 yr**	**3 yr**	**4 yr**	**5 yr**	

Launois et al. 1981 [21]	124 patients March 1973-June 1976 France, single centre, squamous cell	67	64 – 90 Gy preop RT + esophagectomy	4.5	46	20	15	14	10	perioperative mortality was 23% in both groups.
		versus	versus	versus	versus					
		57	esophagectomy (left thoracotomy)	8.2 (mean)	50	35	25	20	12	
					p = NS, but NR					
Gignoux et al. 1987 [22]	229 patients [dates not reported] EORTC, 8 centres, squamous cell, no cervical lesions, no previous cancer, no previous treatment.	115	33 Gy preop RT + esophagectomy	12.3	55	24	20	17	10	tracheosophageal fistula, 2; bleeding, 1; esophagitis, 1; respiratory deaths, 6
		versus	versus	versus	versus					versus
		114	esophagectomy	12 (mean)	57	30	14	11	9	respiratory deaths, 8
					No difference in survival (p = 0.94), but RT may delay local recurrence					
Wang et al. 1989 [23]	206 patients June 1977-May 1985 China, single centre histology not reported < 65 years age, < 8 cm length no metastases	104	40 Gy preop RT + esophagectomy	NR	-	-	-	-	35	leaks, 1; perioperative deaths, 5
		versus	versus	versus	versus					versus
		102	esophagectomy	NR	-	-	-	-	30	leaks, 5; perioperative deaths, 5
					No difference in survival (p > 0.05).					
Nygaard* 1992 [24]	108 patients Jan 1983-Jan 1988 Scandinavia, multi centre squamous cell < 75 years of age, Karnofsky score > 50, T1, T2, Nx, M0 > 21 cm from incisors	58	35 Gy preop RT + esophagectomy	10	44	25	21	-	-	respiratory, 5; leaks, 2; postoperative deaths, 4
		versus	versus	versus	versus					versus
		50	esophagectomy	7	34	13	9	-	-	respiratory, 5; leaks, 2; postoperative deaths, 5
					No difference in survival (p = 0.08).					
Arnott 1992 [25]	176 patients 1979–1983 Scotland, single centre < 80 years, squamous cell adenocarcinoma, distal 2/3 esophagus	90	20 Gy preop RT + esophagectomy	8	40	22	13	9	9	respiratory, 10; postoperative deaths, 10
		versus	versus	versus	versus					versus
		86	esophagectomy (left thoracoabdominal)	8	40	28	23	21	17	respiratory, 5; postoperative deaths, 8; surgical, 2
					No difference in survival (p = 0.40).					
Fok* 1994 [5]	79 patients 1968–1981 Hong Kong, single centre Squamous cell, middle 1/3 esophagus	40	24–53 Gy preop RT + esophagectomy	11	42	34	24	10	10	respiratory, 20; postoperative deaths, 12; leaks 11
		versus	versus	versus	versus					versus
		39	esophagectomy (right thoracotomy, left neck, and abdomen)	22	58	36	24	16	16	respiratory, 15; postoperative deaths, 3; leaks, 7
					No difference in survival.					

*Patients randomized to four groups; data shown are for radiotherapy + surgery versus surgery alone.

Note: EORTC, European Organization for Research and Treatment of Cancer.

**Table 3:** Randomized trials of surgery and postoperative radiotherapy (RT) versus surgery alone.

**Study, year [Reference]**	**Participants**	**Number of patients**	**Interventions**	**Median Survival (Months)**	**Survival Rate (%)**					**Adverse Effects (Number of Patients)**
					**1 yr**	**2 yr**	**3 yr**	**4 yr**	**5 yr**	

Fok et al. 1993 [27]	130 patients July 1986-Dec 1989 Hong Kong, single centre squamous cell adenocarcinoma excluded leaks, respiratory failure, poor performance, metastases	65	esophagectomy + 49–52.5 Gy postop RT	8.7	34	18	16	16	-	gastritis, 6; ulcer, 17; tracheo-esophageal fistulae, 1; strictures, 6
		versus	versus	versus	versus					versus
		65	esophagectomy (Lewis-Tanner or transhiatal or sternal split)	15.2	65	25	21	16	-	gastritis, 3; ulcer, 1; tracheo-esophageal fistulae, 0; strictures, 6
					Shorter survival with RT (p = 0.02). Better local control with RT (p = 0.06) but with more complications.					
Teniere et al. 1991 [28]	221 patients Dec 1979-Dec 1985 France, multi centre squamous cell distal 2/3 esophagus	102	esophagectomy + 45–55 Gy postop RT	18	68	50	27	24	21	minor, 18; major, 4; death, 1
		versus	versus	versus	versus					versus
		119	esophagectomy (transhiatal or right thoracotomy with stomach or colon interposition)	18	73	51	29	22	19	none reported
					No difference in survival (p-value not reported). Local or regional recurrence was lower with RT (70% versus 85%, p-value not reported).					
Fok* 1994 [5]	79 patients 1968–1981 Hong Kong, single centre Squamous cell middle 1/3 esophagus	42	esophagectomy (one or two stage) + 45–53 Gy postop RT	11	48	17	17	12	10	respiratory 25; postoperative deaths 3; leaks 11
		versus	versus	versus	versus					versus
		39	esophagectomy (right thoracotomy, left neck, and abdomen)	22	58	36	24	16	16	respiratory 15; postoperative deaths 3; leaks 7
					No difference in survival.					
Zieren et al. 1995 [29]	68 patients (did not accrue entire sample size 68/160) June 1988-Dec 1991 Germany, single centre squamous cell excluded cervical location, metastases, other cancers, previous treatment	33	esophagectomy + 55.8 Gy postop RT	14	57	29	22	-	-	tracheo-esophageal fistulae, 1; skin, 18; strictures, 2
		versus	versus	versus	versus					versus
		35	esophagectomy (transhiatal or right thoracotomy with stomach interposition)	13	53	31	20	-	-	strictures, 1
					No difference in survival (p-value not reported).					
Xiao et al. 2003 [47]	495 patients	220	Midplane dose of 50–60 Gy in 25–30 fractions over 5–6 weeks	NR	-	-	-	-	41	NR
		versus	versus	versus	versus					
		275	Surgery alone	NR	-	-	-	-	32	
					p = 0.4474					

Note: NR, not reported.

*Patients randomized to four groups; data shown are for surgery + radiotherapy versus surgery alone.

**Table 4:** Randomized trials of preoperative chemotherapy (CT) and surgery versus surgery alone.

**Study, year [Reference]**	**Participants**	**Number of patients**	**Interventions**	**Median Survival (Months)**	**Survival Rate (%)**					**Adverse Effects (Number of Patients)**
					**1 yr**	**2 yr**	**3 yr**	**4 yr**	**5 yr**	

Nygaard* et al. 1992 [24]	106 patients Jan 1983–Jan 1988 Scandinavia, multi centre squamous cell < 75 years of age Karnofsky score > 50 T1, T2, Nx, M0 > 21 cm from incisors	56	cisplatin 20 mg/m^2 ^× 5 days × 2 cycles bleomycin 10 mg/m^2 ^× 5 days × 2 cycles + esophagectomy	7	31	6	3	-	-	respiratory, 3; leaks, 3; postoperative deaths, 6; hematologic, 1; alopecia, 1
		versus	versus	versus	versus					versus
		50	esophagectomy (laparotomy and right thoracotomy)	7	34	13	9	-	-	respiratory, 5; leaks, 2; postoperative deaths, 5
					No difference in survival (p-value not reported).					
Schlag 1992 [32]	46 patients dates not reported Germany, single centre squamous cell < 68 years of age Karnofsky > 70 Stage I, II, III	22	cisplatin 20 mg/m^2 ^× 5 days × 3 cycles 5-fluorouracil 1 g/m^2 ^× 5 days × 3 cycles + esophagectomy	7.5	20	-	-	-	-	vomiting, 11; alopecia, 10; fever, 2; bone marrow suppression, 5; renal, 2;
		versus	versus	versus	versus					versus
		24	esophagectomy (abdominothoracic or thoracoabdominocervical with gastric or colon interposition)	5	32	-	-	-	-	not reported
					No difference in survival (p = 0.91).					
Maipang et al. 1994 [33]	46 patients Aug 1988–Dec 1990 Thailand, single centre squamous cell < 75 years of age ECOG 1, 2. Stage I, II, III distal 2/3 esophagus	24	cisplatin 100 mg/m^2 ^× 1 day × 2 cycles vinblastine 3 mg/m^2 ^× 4 days × 2 cycles bleomycin 10 mg/m^2 ^× 5 days × 2 cycles + esophagectomy	17	58	31	31	-	-	hematologic, 15; vomiting, 15; alopecia, 14; hepatic, 3; lung, 1; urologic, 8; perioperative deaths, 4
		versus	versus	versus	versus					versus
		22	esophagectomy (laparotomy, right thoracotomy with gastric or colon interposition)	17	85	40	36	-	-	none reported
					p = 0.186 Early survival better in surgery alone group.					
Law et al. 1997 [34]	147 patients Dec 1989–Jan 1995 Hong Kong, single centre squamous cell exclude non regional nodes, tracheal involvement, metastases	74	cisplatin 100 mg/m^2 ^× 1 day × 2 cycles 5-fluorouracil 500 mg/m^2 ^× 5 days × 2 cycles + esophagectomy	16.8	60	44	38	28	28	Anemia, 47; neutropenia, 43; thrombocytopenia, 12; renal, 24; vomiting, 34; electrolytes, 21; leaks, 3; pulmonary, 10; respiratory failure, 14; perioperative deaths, 5
		versus	versus	versus	versus					versus
		73	esophagectomy (transhiatal or Lewis-Tanner)	13	50	31	14	14	-	pulmonary, 11; respiratory failure, 22; perioperative deaths, 6
					p = 0.17 Responders to CT lived longer but non-responders had lower median survival than controls (p = 0.03). Lower local recurrence with CT.					
Kok et al. 1997 [35] [abstract]	160 patients 1990–1996 Netherlands, multi-centered Squamous cell	74	cisplatin 80 mg/m^2 ^× 1 day × 2 cycles, etoposide 100 mg IV × 2 days + 200 mg/m^2 ^PO × 2 days × 2 cycles + esophagectomy Note: CT responders received an additional 2 cycles of CT prior to surgery while non-responders received only 2 cycles	18.5						toxic deaths, 1; alopecia, 67; renal, 10
		versus	versus	versus						versus
		74	esophagectomy (transhiatal).	11						none reported
					Not reported but median survival favoured CT (p = 0.002).					
MRC OE02 2002 [37]	802 patients Mar 1992 to June 1998 United Kingdom, multi-centered Resectable esophageal cancer 67% adenocarcinoma, 33% squamous or undifferentiated.	400	cisplatin 80 mg/m^2 ^× 1 day × 2 cycles 5-fluorouracil 1 g/m^2 ^× 4 days × 2 cycles + esophagectomy	16.8	59	43	35	28	26	postoperative complications, 41%; postoperative deaths, 10%
		versus	versus	versus	versus					versus
		402	esophagectomy	13.3	54	34	27	20	15	postoperative complications, 42%; postoperative deaths, 10%
					Significant improvement in survival with chemotherapy HR = 0.79 (95% CI 0.67 to 0.93; p = 0.004)					
Ancona et al. 2001 [48]	94	47	5-FU 1000 mg/m^2 ^CI d1-5 + Cisplatin 100 mg/m^2 ^d1	25	75	55	44	42	34	Gr. 3–4 neutropenia; 10 pts.
		versus	versus	versus	versus					versus
		47	Surgery alone	24	75	55	41	38	22	NR

Note: NR, not reported.

* Patients randomized to four groups; data shown are for chemotherapy + surgery versus surgery alone.

**Table 5:** Randomized trials of preoperative chemotherapy (CT) and postoperative chemotherapy (CT) versus surgery alone.

**Study, year [Reference]**	**Participants**	**Number of patients**	**Interventions**	**Median Survival (Months)**	**Survival Rate (%)**					**Adverse Effects (Number of Patients)**
					**1 yr**	**2 yr**	**3 yr**	**4 yr**	**5 yr**	

Roth et al. 1988 [31]	39 patients	19	cisplatin 120 mg/m^2 ^× 1 day × 1 cycle vindesine 3 mg/m^2 ^× 4 days × 2 cycles bleomycin 10 U/m^2 ^× 4 days × 2 cycles + esophagectomy + cisplatin 120 mg/m^2 ^q 6 wks × 6 months + vindesine 3 mg/m^2 ^q 12 wks × 6 months	9	50	28	28	-	-	alopecia, 17; vomiting, 2; pneumonia, 1; sepsis, 1; neurological, 1; respiratory failure, 1; renal, 1; leaks, 1; chylothorax, 3; pulmonary embolus, 1; wound infection, 1
	Nov 1982–May 1986 NCI, single centre squamous cell Stage I, II, III	versus	versus	versus	versus					versus
		20	esophagectomy (transthoracic with cervical or thoracic anastomosis)	9	35	15	8	-	-	leaks, 3; chylothorax, 1; pulmonary embolus, 1; pneumonia, 1; strictures, 1; empyema, 1; subphrenic abscess, 1
					No difference in survival (p = 0.34). Survival advantage in responders and if less than 10% weight loss.					
Kelsen et al. 1998 [36]	467 patients Aug 1990 to Dec 1995 North America, multi-centered Resectable esophageal cancer 55% adenocarcinoma 45% squamous cell	233	cisplatin 100 mg/m^2 ^× 1 day × 3 cycles 5-fluorouracil 1 g/m^2 ^× 5 days × 3 cycles + esophagectomy + cisplatin 75 mg/m^2 ^× 1 day × 2 cycles if responded	14.9	59	35	23	19	18	minor, 49; major, 53; toxic deaths, 9; neutropenia, 68; mucositis, 58; postoperative deaths, 10
		versus	versus	versus	versus					versus
		234	esophagectomy	16.1	60	37	26	21	20	minor, 67; major, 57; postoperative deaths, 13
					No survival difference.					

Note: NCI, National Cancer Institute

**Table 6:** Randomized trials of surgery and postoperative chemotherapy (CT) versus surgery alone.

**Study, year [Reference]**	**Participants**	**Number of patients**	**Interventions**	**Median Survival (Months)**	**Survival Rate (%)**					**Adverse Effects (Number of Patients)**
					**1 yr**	**2 yr**	**3 yr**	**4 yr**	**5 yr**	

Pouliquen et al. 1996 [39]	120 patients total 62 had curative resections (no residual disease) France, 15 centres July 1987–Mar 1992 Excluded tracheal fistula, >30% liver metastases, brain metastases, node negative resections	24	esophagectomy + cisplatin 100 mg/m^2 ^× 1 day × 6–8 cycles 5-fluorouracil 1000 mg/m2 × 5 days × 6–8 cycles	20	83	34	32	18	17	For 120 patients: tracheoesophageal fistulae, 9; sepsis, 5; infections, 11; pulmonary, 13; gastrointestinal, 26; neurologic, 9; neutropenia, 11; thrombocytopenia, 9; renal, 15; deaths, 4.
		versus	versus	versus	versus					versus
		38	esophagectomy	20	70	44	32	20	12	tracheoesophageal fistulae, 8; sepsis, 4; infections, 9; pulmonary, 12; gastrointestinal, 18; neurologic, 1; neutropenia, 3; thrombocytopenia, 5; renal, 1; no deaths.
					This analysis based only on complete resections. No difference in survival (p-value not reported).					
Ando et al. 1997 [40]	205 patients Japan, multicenter Dec 1988–July 1991 Resectable T1b, < 75 years	105	esophagectomy + cisplatin 70 mg/m^2 ^× 1 day × 2 cycles vindesine 3 mg/m^2 ^× 2 days × 2 cycles	57	90	67	58	58	48	anemia, 2; neutropenia, 13; vomiting, 13; renal, 8; diarrhea, 2; infection, 1.
		versus	versus	versus	versus					versus
		100	esophagectomy (laparotomy and right thoracotomy with 3 field radical lymphadenectomy with gastric or colon interposition).	47	90	67	54	48	45	none reported
					No difference in survival (p = 0.60).					Note: 36% unable to complete chemotherapy due to complications.
Ando et al. 1999 [41] [abstract]	242 patients	120	esophagectomy + cisplatin 80 mg/m^2 ^× 2 cycles 5-fluorouracil (800 mg/m^2 ^× 5 days × 2 cycles	NR	-	-	-	-	51	Grade 3 or 4 hematologic or non-hematologic toxicities were limited in the chemotherapy group.
	Japan, multicenter Jul 1992–Jan 1997	versus	versus	versus	versus					
		122	esophagectomy	NR	-	-	-	-	61	
					No difference in survival (p = 0.30)					

**Table 7:** Randomized trials of preoperative chemoradiation (CRT) and surgery versus surgery alone.

**Study, year [Reference]**	**Participants**	**Number of patients**	**Interventions**	**Median Survival (Months)**	**Survival Rate (%)**					**Adverse Effects (Number of Patients)**
					**1 yr**	**2 yr**	**3 yr**	**4 yr**	**5 yr**	

Nygaard* et al. 1992 [24]	103 patients Jan 1983–Jan 1988 Scandinavia, multi centre squamous cell, < 75 years of age, Karnofsky score > 50, T1, T2, Nx, M0 > 21 cm from incisors	53	cisplatin 20 mg/m^2 ^× 5 days × 2 cycles; bleomycin 5 mg/m^2 ^× 5 days × 2 cycles + 35 Gy sequential radiotherapy + esophagectomy	7	39	23	17	-	-	leaks,2; respiratory, 10
		versus	versus	versus	versus					versus
		50	esophagectomy (laparotomy and right thoracotomy)	7	34	13	9	-	-	respiratory, 5; leaks, 2; postoperative deaths, 5.
					No difference in survival (p = 0.30).					
Le Prise et al. 1994 [42]	86 patients (stopped early after 104/150 patients entered) Jan 1988–April 1991 France, single centre squamous cell, < 70 years of age, < 15% weight loss excluded poor performance, metastases, tracheoesophageal fistula	41	cisplatin 100 mg/m^2 ^× 1 day × 2 cycles 5-fluorouracil 600 mg/m^2 ^× 4 days × 2 cycles + 20 Gy concurrent RT + esophagectomy	11	47	27	19	-	-	Neurological, 1; hematological, 7; renal, 2; tracheo-esophageal fistulae, 3; infections, 4; effusions, 2; deaths, 3; pulmonary embolism, 1; respiratory failure, 1.
		versus	versus	versus	versus					versus
		45	esophagectomy	11	47	33	14	-	-	tracheoesophageal fistulae, 5; infections, 7; effusions, 3; deaths, 3.
					No difference in survival (p = 0.56 at one year).					
Apinop et al. 1994 [43]	69 patients Thailand, single centre Jan 1986–Dec 1992 squamous cell carcinoma Mid to distal 1/3 esophagus, operable	35	cisplatin 100 mg/m^2 ^× 1 day × 2 cycles 5-fluorouracil 1000 mg/m^2 ^× 8 days × 2 cycles + 40 Gy concurrent radiotherapy + esophagectomy	9.7	49	30	26	24	24	leaks, 1; toxic deaths, 2; respiratory, 2; esophageal perforation, 1; cardiovascular, 2; electrolytes, 2
		versus	versus	versus	versus					versus
		34	esophagectomy (right thoracotomy)	7.4	39	23	20	19	10	leaks, 2; respiratory, 2; cardiovascular, 1
					No overall survival difference (p = 0.40 for median survival). Responders had improved survival (p = 0.001).					
Walsh et al. 1996 [44]	113 patients (closed early after 113/190 patients) May 1990–Sept 1995 Ireland, single centre adenocarcinoma < 76 years of age excluded poor performance, metastases, other cancers, previous chemotherapy or radiotherapy	58	cisplatin 75 mg/m^2 ^× 1 day × 2 cycles; 5-fluorouracil 15 mg/kg × 5 days × 2 cycles + 40 Gy concurrent RT + esophagectomy	16	52	37	32	-	-	gastrointestinal, 4; hematologic, 2; cardiac, 15; toxic deaths, 1; respiratory, 28; leaks, 2; recurrent laryngeal nerve palsy, 1; chylothorax, 1
		versus	versus	versus	versus					versus
		55	esophagectomy (transhiatal, or Lewis-Tanner, or abdominal and left thoracotomy)	11	44	26	6	-	-	leaks, 2; recurrent laryngeal nerve palsy, 1; chylothorax, 1; respiratory, 32; cardiac, 13
					Preoperative chemoradiation + surgery prolongs survival compared with surgery alone (p = 0.01). Inferior results in surgery alone arm.					
Bosset et al. 1994 [45]	282 patients Jan 1989–June 1995 France, multi centre squamous cell < 70 years of age < 15% weight loss < WHO status 2 resectable Exclude tracheal fistula, T3N1, T4N0, T4N1	143	cisplatin 80 mg/m^2 ^× 3 days × 2 cycles + 37 Gy concurrent radiotherapy + esophagectomy	18.6	69	48	39	35	33	vomiting, 37; neutropenia, 3; toxic deaths, 1; postoperative deaths, 17; respiratory failure, 6; sepsis,7
		versus	versus	versus	versus					versus
		139	esophagectomy (right thoracotomy + cervical anastomosis)	18.6	67	43	37	34	32	sepsis, 2; postoperative deaths, 5 Note: Trial stopped early 282/320 due to increased mortality in CRT group.
					No difference in overall survival (p = 0.78).					
Urba et al. 2001 [46]	100 patients 1989–1994 Michigan, single centre 25% squamous cell 75% adenocarcinoma	50	cisplatin 20 mg/m^2 ^× 5 days × 2 cycles vinblastine 1 mg/m^2 ^× 4 days × 2 cycles 5-fluorouracil 300 mg/m^2 ^× 21 days + 45 Gy concurrent radiotherapy +esophagectomy	17.6	72	42	30	25	20	grade 3/4 granulocytopenia, 38; grade 3/4 thrombocytopenia, 15; neutropenic fever, 19; red blood cell transfusion, 8; feeding tube, 31; perioperative deaths, 1
		versus	versus	versus	versus					versus
		50	esophagectomy (transhiatal with cervical anastomosis)	16.9	58	38	16	14	10	perioperative deaths, 2; anastomotic leaks, 7 versus 5
					No difference in overall survival (p = 0.15).					
Burmeister et al. 2002 [51]	256 randomized	128†	Cisplatin 80 mg/m^2 ^d1 + 5-FU 800 mg/m^2 ^d2-5 + RT 35 Gy in 15 fractions	22	NR	NR	NR	NR	NR	
		versus	versus	versus	versus					Treatment related mortality 4.6%
		128†	Surgery alone	19	NR	NR	NR	NR	NR	
Lee J-L et al. 2003 [53] [abstract]	102 March 1999 – May 2002 Stage II/III resectable esophageal SCC	52	Cisplatin 60 mg/m^2^ IV d1, 5FU 1,000 mg/m^2 ^IV d2-5, cisplatin 60 mg/m^2^ IV d22 + RT 45.6 Gy, 1.2 Gy bid d1-28 + surgery 3–4 weeks post RT	28.2	NR	NR	NR	NR	NR	NR
		versus	versus	versus	versus					versus
		50	Surgery alone	27.3	NR	NR	NR	NR	NR	NR
				p = 0.67	p = NS					

Note: NR, not reported; NS, not significant.

*Patients randomized to four groups; data shown are for chemotherapy + radiotherapy + surgery versus surgery alone.

† number of patients randomized into each treatment arm estimated from total number of patients.
