# Supplementary material for: Neoadjuvant or adjuvant therapy for resectable esophageal cancer: a systematic review and meta-analysis
Source: BMC Med. 2004 Sep 24;2:35. doi: 10.1186/1741-7015-2-35 (PMC529457; doi:10.1186/1741-7015-2-35)
Supplement: Additional file 1 [file 1741-7015-2-35-S1.doc]

**Appendix 1 - UICC staging for esophageal cancer*.**

| **Stage** | **T (Primary Tumour)** | **N (Regional Lymph Nodes)** | **M (Distant Metastases)** |
| --- | --- | --- | --- |
| 0 | Tis | N0 | M0 |
| I | T1 | N0 | M0 |
| IIA | T2  T3 | N0  N0 | M0  M0 |
| IIB | T1  T2 | N1  N1 | M0  M0 |
| III | T3  T4 | N1  Any N | M0  M0 |
| IV | Any T | Any N | M1 |
| IVA | Any T | Any N | M1a |
| IVB | Any T | Any N | M1b |

Note: UICC, International Union Against Cancer.

* Further details about this staging system in (62): Sobin LH, Wittekind Ch. (Eds): *TNM Classification of Malignant Tumours*. 5^th^ ed. New York: Wiley-Liss, Inc.; 1997:57.
